# Isotopic Discrimination in the Double-Crested Cormorant (*Phalacrocorax auritus*)

**DOI:** 10.1371/journal.pone.0140946

**Published:** 2015-10-16

**Authors:** Elizabeth C. Craig, Brian S. Dorr, Katie C. Hanson-Dorr, Jed P. Sparks, Paul D. Curtis

**Affiliations:** 1 Field of Zoology and Wildlife Conservation, Cornell University, Ithaca, New York, United States of America; 2 United States Department of Agriculture, Wildlife Services, National Wildlife Research Center, Mississippi Field Station, Mississippi State, Mississippi, United States of America; 3 Department of Ecology and Evolutionary Biology, Cornell University, Ithaca, New York, United States of America; 4 Department of Natural Resources, Cornell University, Ithaca, New York, United States of America; Michigan Technological University, UNITED STATES

## Abstract

The diet-tissue discrimination factor is the amount by which a consumer’s tissue varies isotopically from its diet, and is therefore a key element in models that use stable isotopes to estimate diet composition. In this study we measured discrimination factors in blood (whole blood, red blood cells and plasma), liver, muscle and feathers of Double-crested Cormorants (*Phalacrocorax auritus*) for stable isotope ratios of carbon, nitrogen and sulfur. Cormorants exhibited discrimination factors that differed significantly among tissue types (for carbon and nitrogen), and differed substantially (in the context of the isotopic variation among relevant prey species) from those observed in congeneric species. The Double-crested Cormorant has undergone rapid population expansion throughout much of its historic range over the past three decades, leading to both real and perceived conflicts with fisheries throughout North America, and this study provides an essential link for the use of stable isotope analysis in researching foraging ecology, diet, and resource use of this widespread and controversial species.

## Introduction

The use of stable isotope analysis (SIA) has become widespread in studies of wildlife diet and foraging ecology, as the isotopic value observed in the tissue of an animal reflects that of its diet [1,2]. However, as a consumer assimilates dietary nutrients into its own tissues, the isotopic values of those tissues may deviate from that observed in the original diet; a process called isotopic discrimination. The isotopic value observed in the tissue of a consumer (*δ*X_t_) is equivalent to the isotopic value of its diet (*δ*X_d_) plus the diet-tissue discrimination factor (*Δ*X): *δ*X_t_ = *δ*X_d_ + *Δ*X (equation 1), where X is the stable isotope of interest (e.g., ^13^C, ^15^N, or ^34^S). If we know *Δ*X and can measure *δ*X_t_, we can solve for *δ*X_d_, revealing information about the animal’s diet and foraging behavior. Discrimination factors vary among species and among tissue types within a species [3,4] such that, without knowledge of the magnitude of and variance around species- and tissue-specific discrimination factors, meaningful inferences about diet and foraging ecology cannot be made.

Stable isotope values are discussed here using delta notation: *δ*X = (R_sample_—R_standard_)/R_standard_) x 1000 (equation 2), where X is, for example, ^13^C, ^15^N or ^34^S, and R is the corresponding ratio of heavy to light isotopes (e.g., ^13^C/^12^C, ^15^N/^14^N or ^34^S/^32^S) in either the sample of interest (R_sample_) or an international reference standard (R_standard_). Stable isotope ratios, including *δ* ^13^C, *δ* ^15^N and *δ* ^34^S, have proven useful in identifying diet and resource use in wild animals. *δ* ^13^C values are used in diet studies as an indication of the animal’s foraging environment as they are highly influenced by the type of photosynthesis by which carbon is fixed (i.e., C_3_, C_4_ or CAM photosynthesis) as well as the source of carbon and the photosynthetic environment (terrestrial, aquatic, pelagic, benthic, etc.; [5–7]). *δ* ^13^C values tend not to change substantially with trophic position (i.e., little to no *Δ* ^13^C), and for this reason, information about primary production is largely preserved in *δ* ^13^C values throughout the food web [2]. This small *Δ* ^13^C may be due to carbon isotopic fractionation during assimilation or respiration [8]. Researchers have observed *Δ* ^13^C in a variety of aquatic and terrestrial food webs, and reported a range of *Δ* ^13^C from -3 to 4‰, with averages of 0.2‰ [8], 0.4‰ [9], 0.75‰ [4] and the commonly cited 1‰ [2].

*δ* ^15^N values are often used in examining trophic dynamics within food webs as they exhibit significant *Δ* ^15^N across trophic levels, and can therefore be used as an indicator of relative trophic position [10]. *Δ* ^15^N is generally positive, and is largely attributed to the excretion of isotopically lighter nitrogen in urine (or urate in the case of birds), leaving the isotopically heavier nitrogen within the consumer’s system to be assimilated through tissue growth. As with *Δ* ^13^C, researchers have observed *Δ* ^15^N in a variety of aquatic and terrestrial food webs, and reported a range of *Δ* ^15^N from -1 to 10‰, with averages of 3.2‰ [8], 3.4‰ [9] and 2.75‰ [4].

*δ* ^34^S values are highly influenced by whether sulfur fixation occurred in freshwater or marine environments [11], and, as with carbon, exhibits little to no *Δ* ^34^S among trophic positions, preserving information about the source of sulfur throughout the food web. *Δ* ^34^S has been observed to range from -1 to 2‰, with an average of 0.2‰ [8]. The major dietary source of sulfur is in essential amino acids such as cysteine and methionine, which are generally limited in animal diets and therefore are not often considered to have potential for discrimination [12,13].

Isotopic mixing models are increasingly used to identify diet composition (when components of the diet are isotopically distinct [14]). Because discrimination factors are unknown for most organisms, assumptions about the magnitude and direction of discrimination must often be made for these model approaches. However, slight differences in discrimination factors can lead to meaningful differences in the model estimate of diet composition [15,16], and the predictive strength of these models therefore increases greatly if the species- and tissue-specific discrimination factors are known.

In this study we identify *Δ* ^13^C, *Δ* ^15^N and *Δ* ^34^S in the Double-crested Cormorant (*Phalacrocorax auritus*). The Double-crested Cormorant is a piscivorous colonial waterbird native to North America [17]. Over the past three decades this species has undergone rapid population expansion throughout much of its historic range [17–19], leading to both real and perceived conflicts with fisheries throughout North America [20–25]. Rising interest in the diet and foraging behavior of this species has led investigators to seek new methods of evaluating cormorant resource use. Studies have recently begun to employ SIA to this end [13,26–31]. This study provides an essential link for the use of SIA in researching foraging ecology, diet and resource use of this widespread and controversial species.

## Materials and Methods

This research was conducted under the approval of the US Department of Agriculture, Wildlife Services, National Wildlife Research Center’s (NWRC) Institutional Animal Care and Use Committee (protocol QA-1723). Collection of specimens was permitted under USFWS Federal Fish and Wildlife Permit No. MB019065-6, and Mississippi and Alabama State scientific collecting permits. This study did not involve endangered or protected species. Six wild cormorants (all male) were captured at night roosts in Lubbub Creek, Alabama, and Bluff Lake, Mississippi (MS) between 14 and 21 January 2010. These birds were transported to the captive waterbird facility at the NWRC Field Station in Starkville, MS. Cormorants were fed an *ad libitum* diet of farm-raised channel catfish *Ictalurus punctatus* beginning the day after capture for a period of six or eight weeks (n = 3 and n = 3 respectively). Cormorants were sacrificed after the allotted feeding period, allowing for the collection of liver, blood (whole blood, red blood cells and plasma) and feather (with active blood supply) samples. Samples of the catfish diet were also collected throughout the course of the study (n = 22). The duration of time on the catfish diet allowed the isotopic turnover of each tissue type discussed above [32–34]. Pectoralis muscle tissue was also collected from sacrificed birds, but as the period of the feeding trial was shorter than the typical complete turnover time for this tissue (four to five times that of liver [33]), the estimates of discrimination from muscle samples were subject to this additional source of error. Muscle data are still presented here because this source of error was considered to be small as birds likely also consumed farmed catfish prior to capture.

### Stable Isotope Analysis

All samples were frozen and transported to Cornell University for SIA preparation. Feather samples were rinsed with deionized water and dried. Muscle and liver samples were rinsed and drained of blood. Muscle, liver, blood and catfish samples were freeze dried. Muscle, liver and catfish samples were ground to a powder using a freeze mill. Half of each ground muscle, liver and catfish sample was set aside for *δ* ^34^S and *δ* ^15^N analysis without lipid extraction. Due to the influence of lipid content on *δ* ^13^C in cormorant muscle and liver tissue [28], lipids were extracted from the remainder of the cormorant muscle and liver tissues, as well as from the catfish samples, for *δ* ^13^C analysis. Lipids were extracted using a 2:1 chloroform:methanol solvent [35].

All non-lipid extracted samples were analyzed for *δ* ^15^N at the Cornell University Stable Isotope Laboratory (COIL) using a Thermo Finnigan Delta V Advantage isotope ratio mass spectrometer interfaced to a NC2500 elemental analyzer (EA-IRMS; Thermo Scientific, Waltham, MA). Feather, blood, and lipid-extracted muscle, liver and catfish samples were analyzed for *δ* ^13^C at COIL using EA-IRMS. An internal laboratory standard of American mink (*Neovison vison*) tissue was analyzed for every 10 study samples. A chemical methionine standard was used to measure instrumental accuracy across a gradient of amplitude intensities. Isotope corrections were performed using a 2-point normalization (linear regression) of all raw *δ* ^13^C and *δ* ^15^N data with two additional in-house standards: Cayuga Lake brown trout (*Salmo trutta*) and corn (*Zea mays*). Based on standard deviations of within-run replicate measurements of standards, analytical error was estimated to be ± 0.1‰ for *δ* ^13^C, and ± 0.4‰ for *δ* ^15^N. All non-lipid extracted samples were analyzed for *δ* ^34^S at the University of Utah’s Stable Isotope Ratio Facility for Environmental Research using EA-IRMS. Internal laboratory standards were silver sulfide, zinc sulfide and eiderdown, and were analyzed for every 10 study samples. Based on standard deviations of within-run replicate measurements of standards, estimated analytical error was ± 0.5‰ for δ^34^S. Reference standards for ^15^N, ^13^C and ^34^S are atmospheric air, Vienna Pee Dee belemnite and Canyon Diablo troilite respectively.

### Statistical Analysis

All statistical analyses were performed in JMP version 9.0 (SAS Institute 2012). Analysis of variance (ANOVA) with post-hoc Tukey Kramer HSD test was used to identify differences in discrimination factors among tissue types. Statistical analyses were considered significant at *p* < 0.05. Discrimination factors were calculated for each cormorant tissue type by solving for *Δ*X using equation 1, where *δ*X_t_ is the mean isotopic value in each cormorant tissue and *δ*X_d_ is the mean isotopic value of the catfish. Standard deviation for discrimination factors was calculated by adding the standard deviation of isotopic values for each cormorant tissue type to that of the catfish such that it accounted for both sources of variation.

## Results

The catfish diet fed to cormorants in this study had an average (± one standard deviation) *δ* ^13^C value of -19.1‰ (±0.8‰), *δ* ^15^N value of 7.6‰ (±0.5‰) and *δ* ^34^S value of 2.0‰ (±2.3‰) and consisted of 44.2% C (±5.2%), 10.3% N (±1.0%), and 0.6% S (±0.1%). *Δ* ^15^N values were all positive and generally large (ranging from 2.9 to 4.8‰). *Δ* ^13^C values were smaller in magnitude (ranging from -1.1 to 0.2‰) and were predominantly negative. Similar to *Δ* ^13^C, *Δ* ^34^S values were small in magnitude (ranging from -1.1 to 0.5‰; Table 1).

**Table 1:** Average diet-tissue discrimination factors (mean *Δ* ± SD of cormorant tissue + catfish diet) for carbon, nitrogen, and sulfur stable isotopes (*Δ* ^13^C, *Δ* ^15^N, and *Δ* ^34^S) in wild Double-crested Cormorants (*Phalacrocorax auritus*) fed a diet of farm-raised catfish in captivity. Tissue types that differed significantly from one another (*p* < 0.05) are indicated by different capital letters after parentheses within each column (ANOVA with post-hoc Tukey Kramer HSD).

Tissue	Discrimination factor (mean ± SD)		
	*Δ* ^13^C (‰)	*Δ* ^15^N (‰)	*Δ* ^34^S (‰)
Feather	0.2 ± 1.3 ^A^	4.8 ± 0.8 ^A^	-0.1 ± 4.1 ^A^
Liver	-0.3 ± 1.1 ^AB^	4.3 ± 0.5 ^B^	-0.9 ± 3.8 ^A^
Muscle ^a^	-1.1 ± 1.2 ^C^	3.9 ± 0.7 ^C^	-1.1 ± 3.4 ^A^
Plasma	-0.6 ± 1.1 ^BC^	3.9 ± 0.7 ^BC^	-0.5 ± 4.1 ^A^
Red blood cells	-0.8 ± 1.1 ^BC^	3.0 ± 0.6 ^D^	0.1 ± 3.2 ^A^
Whole blood	-1.1 ± 1.1 ^C^	2.9 ± 0.6 ^D^	0.5 ± 2.9 ^A^

^a^Muscle tissue may not have experienced a full isotopic turnover during the captive period, however this source of error was minimized by the fact that birds likely consumed a similar diet prior to capture.

## Discussion

Double-crested Cormorants exhibited discrimination factors that differed significantly among tissue types (for *Δ* ^13^C and *Δ* ^15^N; Table 1). This was expected in light of the diversity of biochemical and physiological processes taking place within these functionally different tissues. While both positive and negative *Δ* ^13^C and *Δ* ^34^S values were observed, all were relatively close (within approximately 1‰) to zero. All discrimination factors observed in this study were within the range of values observed in freshwater aquatic food webs [2,4,8,9,12,13].

Unexpectedly, Double-crested Cormorant discrimination factors differed substantially (in the context of the isotopic variation among relevant prey species) from those observed in Great Cormorants (*P*. *carbo* [36,37]) and European Shags (*P*. *aristotelis* [36]). While *Δ* ^15^N values observed in Double-crested Cormorant tissues were within the range observed in congeneric species, *Δ* ^13^C exhibited inter-specific differences in both magnitude and direction. Double-crested Cormorant *Δ* ^13^C values were small and primarily negative while those observed in the Great Cormorant and European Shag were larger and primarily positive (Table 2). It is likely that some of the variation observed among species is due to differences in experimental design. The current study had a relatively small sample size, sampled only males in captivity, and removed lipids from diet samples. In comparison, Mizutani et al. [37] sampled individuals in captivity, but did not report sex, and did not extract lipids from diet samples (influencing *Δ* ^13^C estimates). Bearhop et al. [36] used a combination of wild (European Shag) and captive (Great Cormorant) birds, did not report sex, but did extract lipids from diet samples. These experimental differences make direct interspecific comparisons of discrimination factors difficult. However, there are also potential biological sources of this variation, including interspecific differences in metabolic rates or physiological processes. For instance, depending on the energy needs of the consumer, carbohydrates and fatty acids from the diet will be routed towards respiration (for immediate energy needs), fatty acid synthesis, or fat storage. Proteins from the diet are broken down, and their composite amino acids are either dissembled or, in the case of essential amino acids, used directly in protein synthesis. These biochemical and physiological processes determine the fate of nutrients consumed in the diet, and therefore play a key role in the magnitude and direction of diet-tissue discrimination.

**Table 2:** Average diet-tissue discrimination factors for carbon (*Δ* ^13^C) and nitrogen (*Δ* ^15^N) in two *Phalacrocorax* species: the Great Cormorant (*P*. *carbo*) and the European Shag (*P*. *aristotelis*), adapted from Hobson [31].

Species	Tissue	n	Discrimination factor (mean)		Source
			*Δ* ^13^C (‰)	*Δ* ^15^N (‰)	
*P*. *carbo*					
	Feather	8	2.3	4.2	Bearhop et al. [36]
	Feather	17	3.8	3.7	Mizutani et al. [37]
	Muscle		1.3	2.4	Mizutani et al. [37]
	Liver		4.2	4.8	Mizutani et al. [37]
*P*. *aristotelis*					
	Feather	12	2.0	3.6	Bearhop et al. [36]

## Conclusions

Because of their substantial differences in magnitude and direction, use of discrimination factors from different species, even congeneric species like the Great Cormorant or European Shag, to model diet in the Double-crested Cormorant would introduce substantial error into model estimates, underlining the importance of using species- and tissue-specific discrimination factors in modeling efforts.
